# Urinary microbiota shift is associated with a decline in renal function

**DOI:** 10.1093/lifemedi/lnad014

**Published:** 2023-04-19

**Authors:** Yonglin Wu, Miaomiao Pan, Zheng Zou, Xingyu Rong, Hao Yang, Zhenming Xiao, Huijing Wang, Tao Liu, Wu Huang, Meifang Shi, Chao Zhao

**Affiliations:** MOE/NHC/CAMS Key Lab of Medical Molecular Virology, School of Basic Medical Sciences and National Clinical Research Center for Aging and Medicine, Huashan Hospital, Shanghai Medical College, Fudan University, Shanghai 200032, China; MOE/NHC/CAMS Key Lab of Medical Molecular Virology, School of Basic Medical Sciences and National Clinical Research Center for Aging and Medicine, Huashan Hospital, Shanghai Medical College, Fudan University, Shanghai 200032, China; Department of Urology, Youyi Road Community Health Service Centre for Baoshan District, Shanghai 201999, China; Department of Medical Chemistry, Graduate School of Medicine, Kyoto University, Kyoto 606-8501, Japan; MOE/NHC/CAMS Key Lab of Medical Molecular Virology, School of Basic Medical Sciences and National Clinical Research Center for Aging and Medicine, Huashan Hospital, Shanghai Medical College, Fudan University, Shanghai 200032, China; MOE/NHC/CAMS Key Lab of Medical Molecular Virology, School of Basic Medical Sciences and National Clinical Research Center for Aging and Medicine, Huashan Hospital, Shanghai Medical College, Fudan University, Shanghai 200032, China; Laboratory of Neuropsychopharmacology, College of Fundamental Medicine, Shanghai University of Medicine and Health Science, Shanghai 201318, China; Department of Urology, Youyi Road Community Health Service Centre for Baoshan District, Shanghai 201999, China; MOE/NHC/CAMS Key Lab of Medical Molecular Virology, School of Basic Medical Sciences and National Clinical Research Center for Aging and Medicine, Huashan Hospital, Shanghai Medical College, Fudan University, Shanghai 200032, China; Department of Urology, Youyi Road Community Health Service Centre for Baoshan District, Shanghai 201999, China; MOE/NHC/CAMS Key Lab of Medical Molecular Virology, School of Basic Medical Sciences and National Clinical Research Center for Aging and Medicine, Huashan Hospital, Shanghai Medical College, Fudan University, Shanghai 200032, China; Shanghai Frontiers Science Center of Pathogenic Microbes and Infection, Shanghai 200032, China

**Dear Editor,**

The aging process affects multiple organ systems and exhibits complex phenotypes. Renal insufficiency during aging can greatly affect homeostasis and inflammation [1]. Renal damage and the accompanying loss of function alter the aging process and affect the incidence and prognosis of various diseases and complications. The molecular mechanisms and histological features of renal aging in general have been extensively explored, notably through the application of emerging research methods. The investigation of urinary symbiotic flora enabled by high-throughput sequencing has revealed that the pathophysiology of many diseases is associated with specific features of the urinary microbiome [2]. However, the lack of diagnostic methods in the field of renal function poses a barrier to the identification of early renal aging and increases the likelihood of poor outcomes for patients. Therefore, given the ease of noninvasive urine collection and specificity of the urinary symbiotic microbiota, we explored the association between changes in the urinary microbiome and early decline in renal function.

We analyzed the associations of blood urea nitrogen (BUN), creatinine (CREA), and the estimated glomerular filtration rate (eGFR) with aging in healthy individuals (*n* = 3,342) undergoing routine medical examinations. Based on our screens for a sharp decline in renal function; the 74 individuals in the 50- to 65-year-old age group were divided roughly equally into the upper quartile, middle quartiles, and lowest quartiles of renal function, and clean-catch midstream urine samples were collected for urinary microbiota 16S rDNA sequencing. The dominant microbiota were determined and their relationships with chronic kidney disease were investigated using an analysis that combined relevant clinical indicators with information from public databases on previously reported associations between microbiota and disease (Fig. S1).

All renal function marker levels showed a clear grouping between the ages of 50 and 65 years (Fig. S2A–C). More significant inter-individual differences were observed between the individuals aged 50–65 years in both the entire cohort and in males and females, and these changes in renal function tended to decline steadily with age (Figs. 1A and S2D). In addition, females generally outperformed males with regard to renal function indicators (Fig. S2E and S2F). There were significant differences in the structure and composition of the urine microbial communities between males and females (Figs. 1B–F and S3A). The dominant enriched microbiota in females were more resistant to antibiotics than the enriched microbiota in the male cohort (Fig. 1G). Thus, the enrichment of potential urologic pathogens in the renal function decline group may be significant.

**Figure 1.**
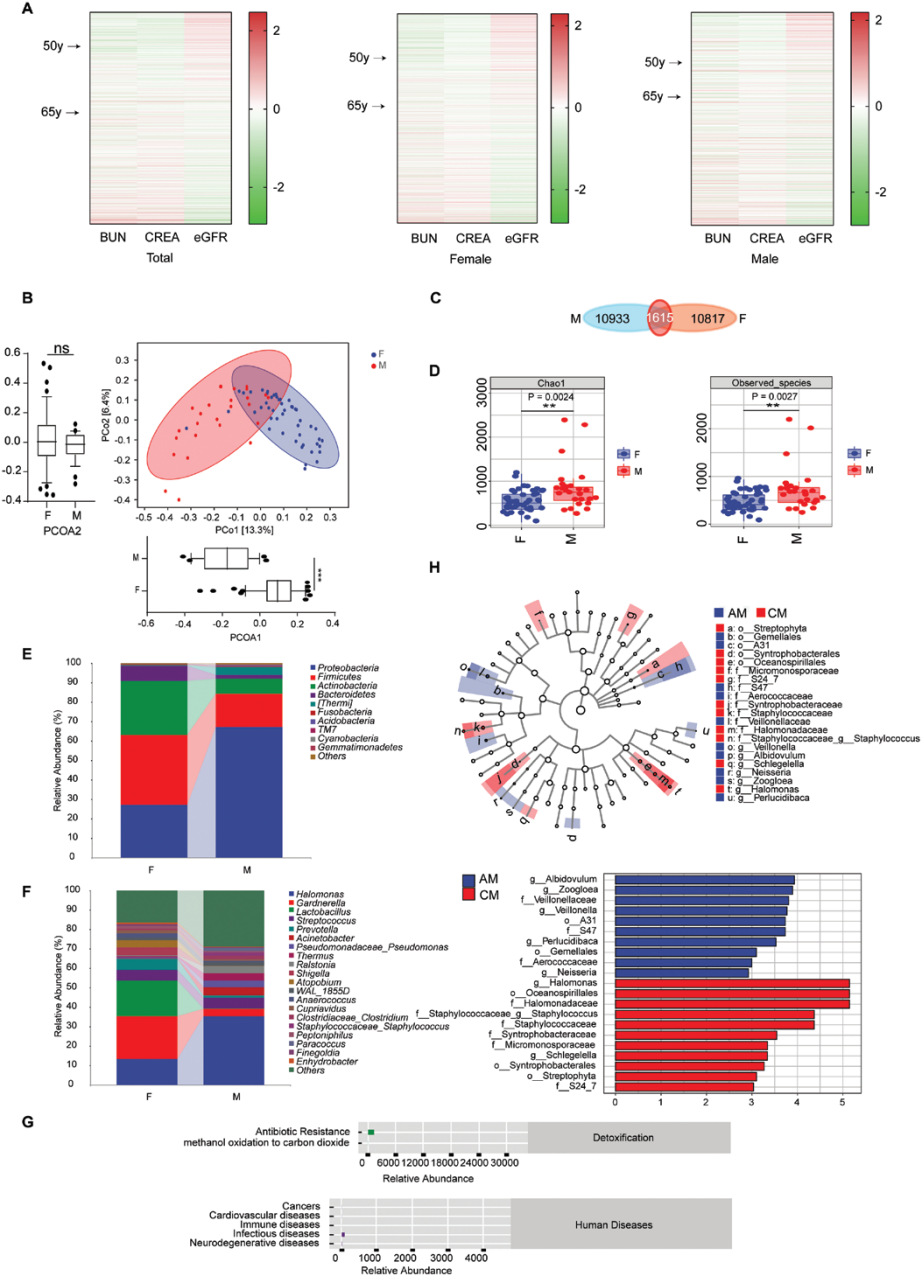
Age-related changes in renal function in healthy individuals and urinary microbiota alterations associated with renal function changes in different cohorts. (A) Heat maps showing the changes in BUN, CREA and eGFR with age in the total population, in females only and in males only. (B) PCoA based on UniFrac distances. Each sample is represented by a dot. The blue circle represents the female group and the red circle represents the male group. PCo1 explained 13.3% of the variation and PCo2 explained 6.4% of the variation in the composition of the microbiota. The box chart shows the degree of difference in the horizontal and vertical coordinates, respectively; ns, *P* > 0.05; ***P < 0.001. (C) Venn diagrams at the OTU level. The two colors represent different groups. The overlapping region represents the common OTUs between groups; the non-overlapping regions represents the unique OTUs in each group. (D) Alpha diversity indices for the female and male groups, including the Chao1 and observed-species indices; **P < 0.01. (E) Major taxa of dominant microbiota at the phylum level. (F) Major taxa of dominant microbiota at the genus level. (G) PICRUSt functional prediction. The ordinate is MetaCyc second-level functional pathway classification. MetaCyc first-level functional pathway classification is shown the right. (H) LEfSe to identify potential species markers. Cladogram of taxonomic representations of significant differences between the AM and CM groups. Coloured nodes from the inner to the outer ring represent taxa from the phylum to genus levels. The two colors represent different taxa. The LDA score threshold for identifying features was 2.0.

Further analysis revealed the β diversity of the urine microbiome was significantly different between the groups of 50–65-year-olds; for example, between males in the top quartile of renal function (AM group) and males in the lower quartile of renal function (CM group) and between females in the top quartile of renal function (AF group) and females in the lower quartile of renal function (CF group; Fig. S3B–D). There were also significant differences in the α diversity of the microbiota between the upper and lower quartiles of females (*P* < 0.05), but not in males (Fig. S3E–F). These results suggest that the urinary microbiota changes with renal aging in both sexes, and that these changes are greater in females. LEfSe analysis showed enrichment of *F_Staphylococcaceae* in the CM group compared to the AM group (LDA score > 2.0, *P* < 0.05). These bacteria have been associated with bladder cancer and urinary tract infections [3], which suggests that age-associated changes in the urinary microbiota may make males more susceptible to bladder cancer and other urinary tract diseases (Fig. 1H). The dominant enriched microbiota also varied between the AF and CF groups (LDA score > 2.0, *P* < 0.05; Fig. S3G). Based on these results, the female urinary microbiota changes with renal aging and these alterations may be related to a rapid decline in renal function. Furthermore, the changes in the microbiota are likely to increase susceptibility to urological diseases and, in turn, these diseases may accelerate renal aging.

To investigate the relationships between the selected dominant microbiota, renal aging, electrolyte imbalances, and inflammation, we selected 14 clinical parameters, including indicators related to electrolyte balance (Na^+^, K^+^, and Ca^2+^) and inflammation (hs-CRP, WBC, NEUT_ per, LYMPH_ per, CHO, HDL-C, HBA1C, ALT, AST, and GGT). Correlation analysis revealed the level of the plasma electrolyte Na^+^ negatively correlated with the dominant microbiota *g_Streptococcus* (*r* = −0.2638, *P* < 0.05) and that Ca^2+^ negatively correlated with *g_Bacillus* (*r* = −0.26, *P* < 0.05) (Fig. 2A). in the group of females with poorest renal function (CF). In analysis of the correlations between the dominant enriched microbiota and clinical indicators related to inflammation (Fig. 2B), the inflammatory markers hs-CRP and WBC were positively correlated with *g_Finegoldia* (*r* = 0.42, *P* < 0.05) and *o_Syntrophobacterales* (*r* = 0.30, *P* < 0.05), respectively, the dominant microbiota in the poor renal function CM and CF groups. These results suggest that the changes in the microbiota that correlate with decreased renal function are associated with the activation of inflammation. Indicators of inflammatory changes in the host, including the glucolipid metabolism-related indicators HBA1C, CHO, and HDL-C and the inflammation-related plasma enzymes AST, ALT, and GGT, positively correlated with the dominant microbiota in the poor renal function group and negatively correlated with the dominant microbiota in the good renal function group. For example, HBA1C was positively correlated with *g_Streptococcus*. In contrast, *g_ Streptococcus* and *g_Bacillus* were positively correlated with adverse alterations in Na^+^ and Ca^2+^, indicators related to the metabolic status of electrolytes in the kidney. These observations further indicate that activation of organismal inflammation plays an important role in the microbiota changes associated with a decline in renal function.

**Figure 2.**
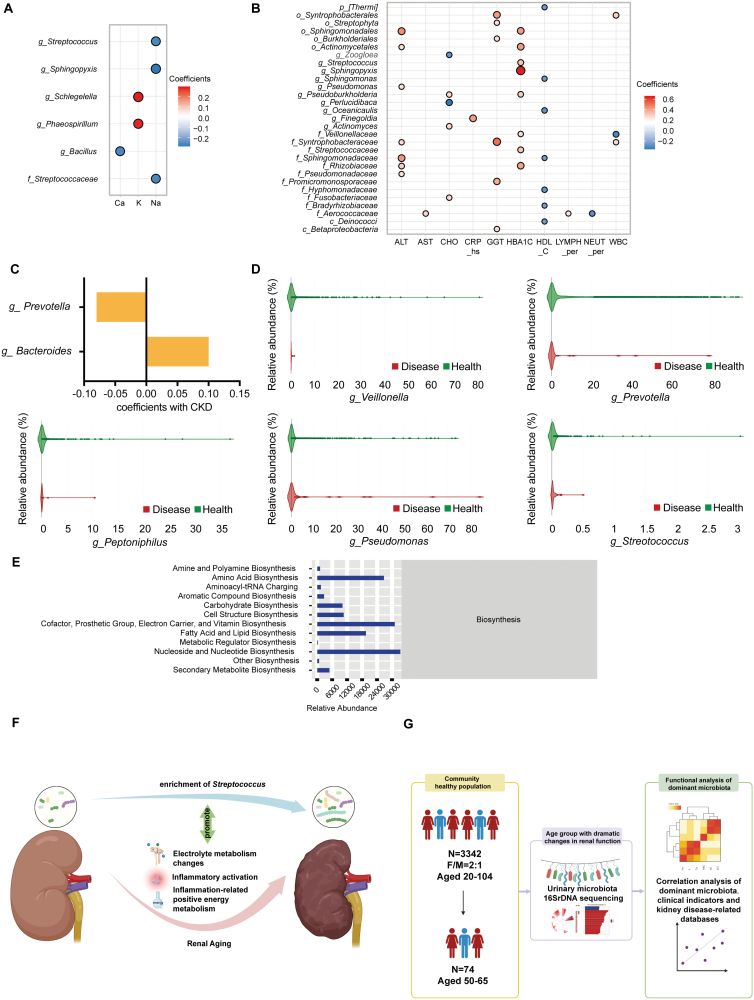
Analysis of the correlations between urinary microbiota and clinical indicators related to homeostasis, inflammation and chronic kidney disease. (A) Scatter plot showing the correlation between changes in dominant microbiota and changes in electrolyte metabolism during renal aging (*P* < 0.05). (B) Scatter plot showing the correlation between changes in the dominant microbiota during renal aging and clinical indicators of aging-related inflammation (*P* < 0.05). (C) Associations *of g_Prevotella* and *o_Bacteroidales* with chronic kidney disease (MicrophenoDB database). (D) Associations of *g_Veillonella*, *g_Streptococcus*, *g_Prevotella*, *g_Peptoniphilus*, and *g_Pseudomonas* with chronic kidney disease (GMrepo database). (E) PICRUSt functional prediction. The ordinate is MetaCyc second-level functional pathway classification. MetaCyc first-level functional pathway classification is shown on the right. (F) Mechanisms by which *Streptococcus* is possibly involved in renal aging. (G) Graphic summary.

The results above indicate the urinary microbiota are significantly altered in both men and women with deterioration of renal function, and these changes are closely related to organismal electrolyte metabolism and inflammatory activation. Therefore, we used microbiota databases to explore the possible mechanisms by which the dominant altered microbiota may promote chronic kidney disease. The dominant microbiota in the AF group, *g_ Prevotella*, is negatively associated with chronic kidney disease in the MicrophenoDB database. In contrast, *g_ Bacteroides*, which belongs to the dominant microbiota *o_Bacteroidales* in the CF group, is positively associated with chronic kidney disease (Fig. 2C). Further analysis using the GMrepo database revealed that the dominant microbiota *g_ Veillonella* and *g_ Prevotella* in the good renal function group are negatively associated with chronic kidney disease, while *g_Peptoniphilus*, *g_Pseudomonas*, and *g_Streptococcus* in the poor kidney function group are positively associated with kidney disease (Fig. 2D). In addition, analysis of the Disbiome database revealed that *f_Prevotellaceae* and *g_Prevotella* in the good kidney function group are negatively associated with chronic kidney disease, while *g_Weissella*, *g_Pseudomonas*, *g_Bacillus*, and *g_Streptococcus* in the poor kidney function group are positively associated with chronic kidney disease [4–8].

Among the urinary microbiota alterations described above, *g_Streptococcus* is closely associated with changes in electrolyte balance and may potentially reflect early renal impairment. In addition, *g_Streptococcus* is also associated with a higher positive energy balance during aging, which may trigger low-grade inflammation. This suggestion is corroborated by the prediction of microbial functional pathways for the AF/CF groups, which showed a significant increase in anabolic pathways such as lipid synthesis (Fig. 2E). Therefore, we suggest that an increased abundance of *g_Streptococcus* in urine may play a critical role in inflammatory renal impairment and may serve as a good predictor of renal aging. Moreover, in view of the strong association between *Streptococcus* and kidney diseases reported elsewhere [9], the occurrence of *g_Streptococcus* in urine could indicate a pathogenic mechanism of renal aging, and the possible mechanisms are summarized in Fig. 2F.

This study suggests an inflection point in the decline in kidney function occurs between the ages of 50 and 60 years. Interestingly, this result is consistent with a previous study of the changes in kidney volume in healthy individuals [10] and suggests that the inflection point in the decline in renal function with age is presumably closely related to both macroscopic and microscopic renal changes. In addition, the dominant bacteria in the CF group have pathogenic potential to cause urinary system diseases and participate in the activation of inflammation. From the perspective of aging-related changes in energy metabolism, many of the dominant microbiota in females and males with the poorest renal function (CF and CM groups) correlate positively with a positive energy balance, with *g_Streptococcus* being one of the more prominent examples. This clade is not only associated with alterations to energy metabolism, but also positively correlated with chronic nephritis. Moreover, the increased expression of the energy anabolic pathway inferred in the CF group further supports the idea that low-grade inflammation induced by a high positive energy balance plays an important role in aging, especially renal aging. Our data therefore support the hypotheses that the microbiota play a role in influencing inflammation and metabolic changes in the cells of the human body and that the microbiota presumably regulate the aging process, at least partly by these mechanisms. Among the altered microbiota identified, *f_ Streptococcaceae* and its subordinate *g_ Streptococcus* may represent good indicators of renal aging in females.

## Research limitations

We observed an evident decline in renal function occurs at a particular age (50–65 years) and identified specific microbiota in the urine as predictive indicators for early renal aging and healthy aging. However, a limitation of this study is that the results of our validation cohort are still being explored through screening for differential bacteria and deep metagenomic-based functional mining would be very meaningful.

## Supplementary Material

lnad014_suppl_Supplementary_Data
