# Supplementary material for: Urinary microbiota shift is associated with a decline in renal function
Source: Life Med. 2023 Apr 19;2(3):lnad014. doi: 10.1093/lifemedi/lnad014 (PMC11749367; doi:10.1093/lifemedi/lnad014)
Supplement: lnad014_suppl_Supplementary_Data [file lnad014_suppl_Supplementary_Data.pdf]

Supplementary Information

Urinary microbiota shift is associated with a decline in renal function

Materials and Methods

Study design and participants

We conducted a cross-sectional study of 3342 healthy subjects (2294 females and 1048 males) aged 20–104 recruited from the Health Services Management Center of Baoshan District in Shanghai. Participants who met the following health criteria were designated as healthy: (1) not obese, i.e., body mass index (weight [Kg]/(height [m])2) <28; (2) no history of chronic kidney disease, cancer, Alzheimer's disease, Crohn's disease, ulcerative colitis, diabetes mellitus, Cushing syndrome, hyperthyroidism, rheumatoid arthritis or other chronic illnesses; (3) no history of hepatitis virus infection; (4) no consumption of anti-inflammatory drugs within the previous 2 weeks or antibiotics within 3 months (based on self-reporting using questionnaires); (5) no alcohol consumption within the previous month; and (6) no history of smoking. The healthy cohort was divided into seven age groups: 20–30, 31–40, 41–50, 51–60, 61–70, 71–80 and 81–100-years-old. Blood was collected from each subject and serum was tested for blood urea nitrogen (BUN), creatinine (CREA) and estimated glomerular filtration rate (eGFR).

After establishing the age range (50–65) in which significant changes in renal function indicators were evident, we conducted a quartile-based comparative renal function analysis for this interval. The portion of the healthy population aged 50–65 with significant changes in renal function was divided into the top quartile with good renal function, two quartiles with medium renal function, and the bottom quartile with poor renal function. A small study cohort was established by randomly selecting 17, 16, and 15 females and nine, eight, and nine males from the three subgroups, respectively, for a total of 74 cases. Clean-catch midstream urine samples were collected for urinary microbiota 16S rDNA sequencing.

Sample collection and clinical laboratory measurements

Before sampling, subjects were informed not to use antibiotics or probiotics for one week. Blood samples were taken from all patients in the morning after they had been seated for 5 minutes. Blood was drawn in EDTA anti-coagulated collection tubes and serum-separator collection tubes by

phlebotomists for routine blood tests and biochemical measurements. High-sensitivity C-reactive protein (hs-CRP), white blood cell count (WBC), neutrophil percentage (NEUT_per) and lymphocyte percentage (LYMPH_per) were evaluated using a Sysmex XN-9000 automatic hematology analyzer. Blood urea nitrogen (BUN), serum creatinine (CREA), estimated glomerular filtration rate (eGFR), serum sodium ion (Na^+), potassium ion (K^+) and calcium ion (Ca^{2+}) concentrations, haemoglobin A1C (HBA1C), cholesterol (CHO), high density lipid-cholesterol (HDL-C), aspartate transaminase (AST), alanine transaminase (ALT) and γ-glutamyl transpeptidase (GGT) were analysed using a Beckman Coulter Chemistry Analyzer AU5800. At the same time, clean midstream urine was also collected and stored at -80 °C.

Urine symbiotic microbe 16S rDNA sequencing

We performed 16S rDNA sequencing on the urine samples collected from the 74 cases. Total genomic DNA samples were extracted using the OMEGA Soil DNA Kit (M5635-02; Omega Bio-Tek, Norcross, GA, USA), following the manufacturer's instructions, and stored at -20 °C prior to further analysis. The quantity and quality of the extracted DNA samples were assessed using a NanoDrop NC2000 spectrophotometer (Thermo Fisher Scientific, Waltham, MA, USA) and agarose gel electrophoresis, respectively. PCR amplification of the bacterial 16S rRNA gene V3–V4 region was performed using the forward primer 338F (5`-ACTCCTACGGGAGGCAGCA-3`) and the reverse primer 806R (5`-GGACTACHVGGGTWTCTAAT-3`). Sample-specific 7-bp barcodes were incorporated into the primers for multiplex sequencing. The PCR components contained 5 μL of buffer (5×), 0.25 μL of Fast pfu DNA Polymerase (5U/μL), 2 μL (2.5 mM) of dNTPs, 1 μL (10 uM) of each forward and reverse primer, 1 μL of DNA template and 14.75 μL of ddH2O. Thermal cycling consisted of initial denaturation at 98 °C for 5 min, followed by 25 cycles of denaturation at 98 °C for 30 s, annealing at 53 °C for 30 s and extension at 72 °C for 45 s, with final extension for 5 min at 72 °C. PCR amplicons were purified with Vazyme VAHTSTM DNA Clean Beads (Vazyme, Nanjing, China) and quantified using the Quant-iT PicoGreen dsDNA Assay Kit (Invitrogen, Carlsbad, CA, USA). After the individual quantification step, amplicons were pooled in equal amounts, and paired-end 2×250 bp sequencing was performed using the Illumina NovaSeq platform with a NovaSeq 6000 SP Reagent Kit (500 cycles) at Shanghai Personal Biotechnology Co., Ltd. (Shanghai, China).

Processing and analysis of sequence data

Primer removal, mass filtration, splicing and chimera removal were carried out using the DADA2 method and assessed using QIIME2. After processing, the sequencing reads were mapped at 97% similarity level using Vsearch (v2.13.4_linux_x86_64). The obtained operational taxonomic unit (OTU) data was then analysed by reference to the Greengenes database (Release 13.8, http://greengenes.secondgenome.com/). To evaluate the phylogenetic relationships among OTUs, we used QIIME2 to call mafft and FastTree to construct a phylogenetic tree. To assess alpha diversity, we used the Chao1 index and observed species index at the OTU level. In order to evaluate beta diversity, we used unweighted UniFrac distance for principal coordinate analysis (PCoA). Venn diagrams based on OTU abundance were used to visualize the differences between groups. The QIIME2 statistical characteristic table was used to analyze species composition at the phylum and genus levels, and stacked histograms were used to visualize the composition distribution. Linear discriminant analysis effect size (LEfSe) was used to find microbial markers that differed among sample groups. LEfSe combines the nonparametric Kruskal-Wallis and Wilcoxon rank sum tests with linear discriminant analysis (LDA) and performs difference analysis under the conditions that the Kruskal-Wallis P-value is less than 0.05 with an LDA score threshold of >2.0. Phylogenetic Investigation of Communities by Reconstruction of Unobserved States 2 (PICRUSt2) was used to predict sample function in the MetaCyc function database (https://metacyc.org/) based on marker gene sequences. R (Version 4.0.0) was used for Pearson correlation analysis of clinical indicators and OTU abundance of samples and Bioinfo Intelligent Cloud (BIC; http://www.ehbio.com/Cloud_Platform) was used for visualization.

Database and epidemiological analysis

The MicrophenoDB (http://www.liwzlab.cn/microphenodb), GMrepo (https://gmrepo.humangut.info/phenotypes/) and Disbiome databases (http://disbiome.ugent.be) were used to analyse the associations between dominant features of the microbiome and kidney disease.

Statistical analysis

Spearman and Pearson correlation analyses were used to evaluate the correlations between biochemical indicators and clinical data. The top and bottom 2.5% ranges were excluded. The Kolmogorov-Smirnov method was used to test the normality of data. The Student's *t*-test, one-way ANOVA and Wilcoxon test were used to evaluate the significance of the differences in the clinical

indicators among groups. A two-tailed P-value <0.05 was considered statistically significant.

Data availability

The datasets generated and/or analysed during the current study are available from the China National Center for Bioinformation portal repository (HRA003367), https://ngdc.cncb.ac.cn/sso/login.

Research ethics

This study was approved by the ethics committee of Shanghai Medical College of Fudan University (ethics committee approval letter No. 2020-C017).

Figure S1

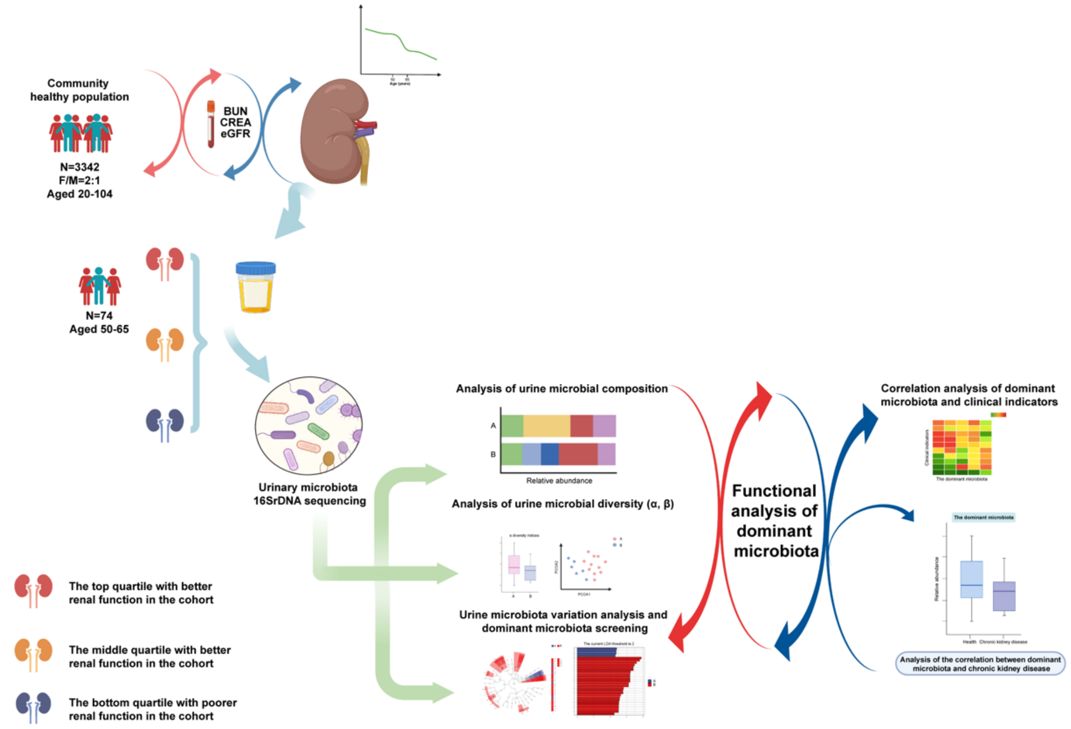

Figure S1. Summary of study design and analysis process.

Changes in renal function with age were analysed in 3342 healthy participants (female: male ratio, 2:1). From these participants, 74 individuals from the age group with dramatic changes in renal function were selected for analysis of urinary microbiota and clinical indicators, in combination with analysis of kidney disease-related databases.

Figure S2

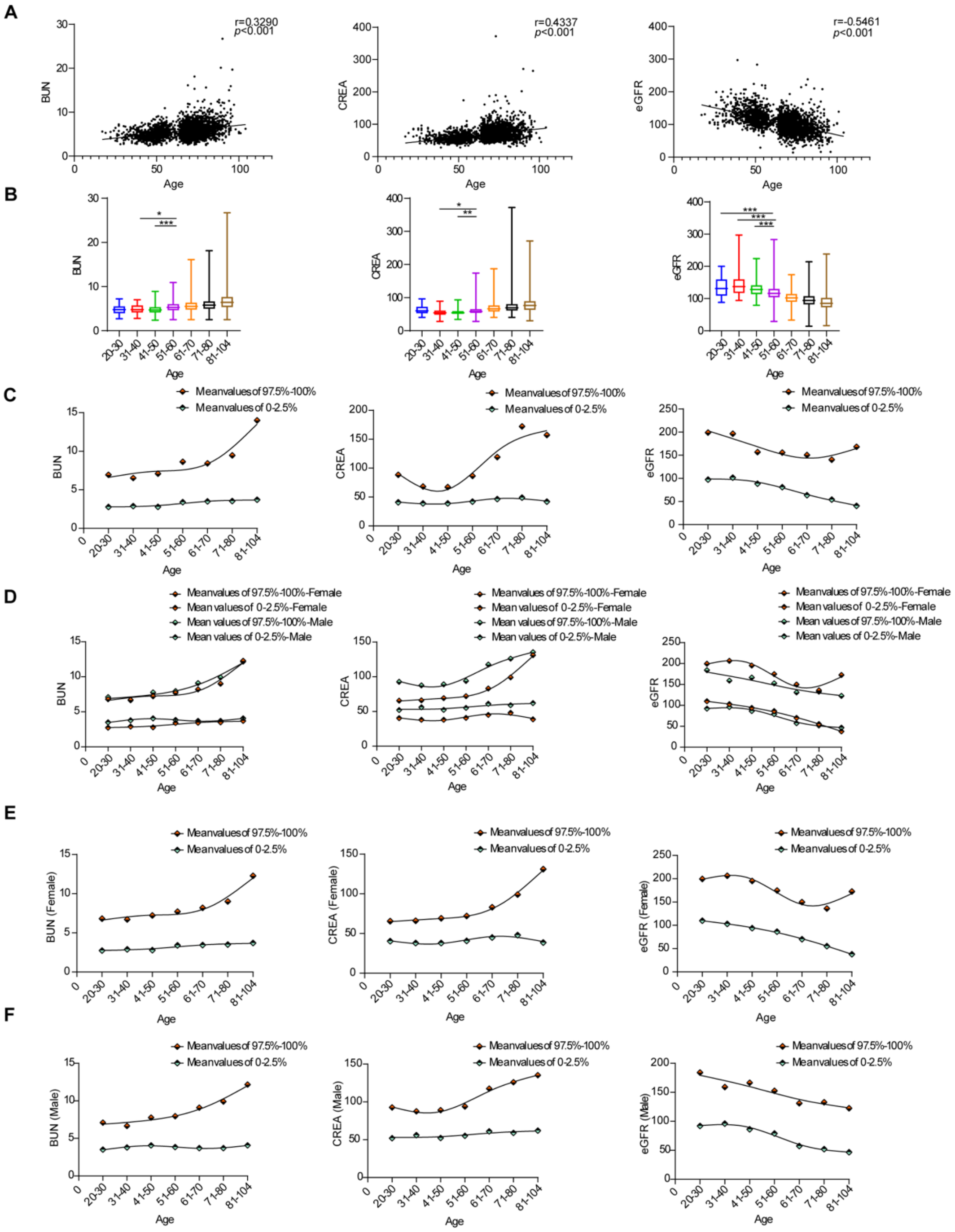

Figure S2. Identification of age-related changes in renal function in a healthy population.

(A) Pearson correlation analysis line graph showing the correlations between age and the clinical indicators of renal function BUN, CREA and eGFR ($P < 0.0001$).

(B) Box plots showing the associations between age and BUN, CREA and eGFR. One-way ANOVA with Bonferroni's test; *, $P < 0.05$; **, $P < 0.01$; ***, $P < 0.001$. (C-F) Comparative analysis of the 2.5th and 97.5th percentiles of BUN, CREA and eGFR for each age group of (C) the total population, (D) combined male and female groups and (E) female groups, and (F) male groups.

Figure S3

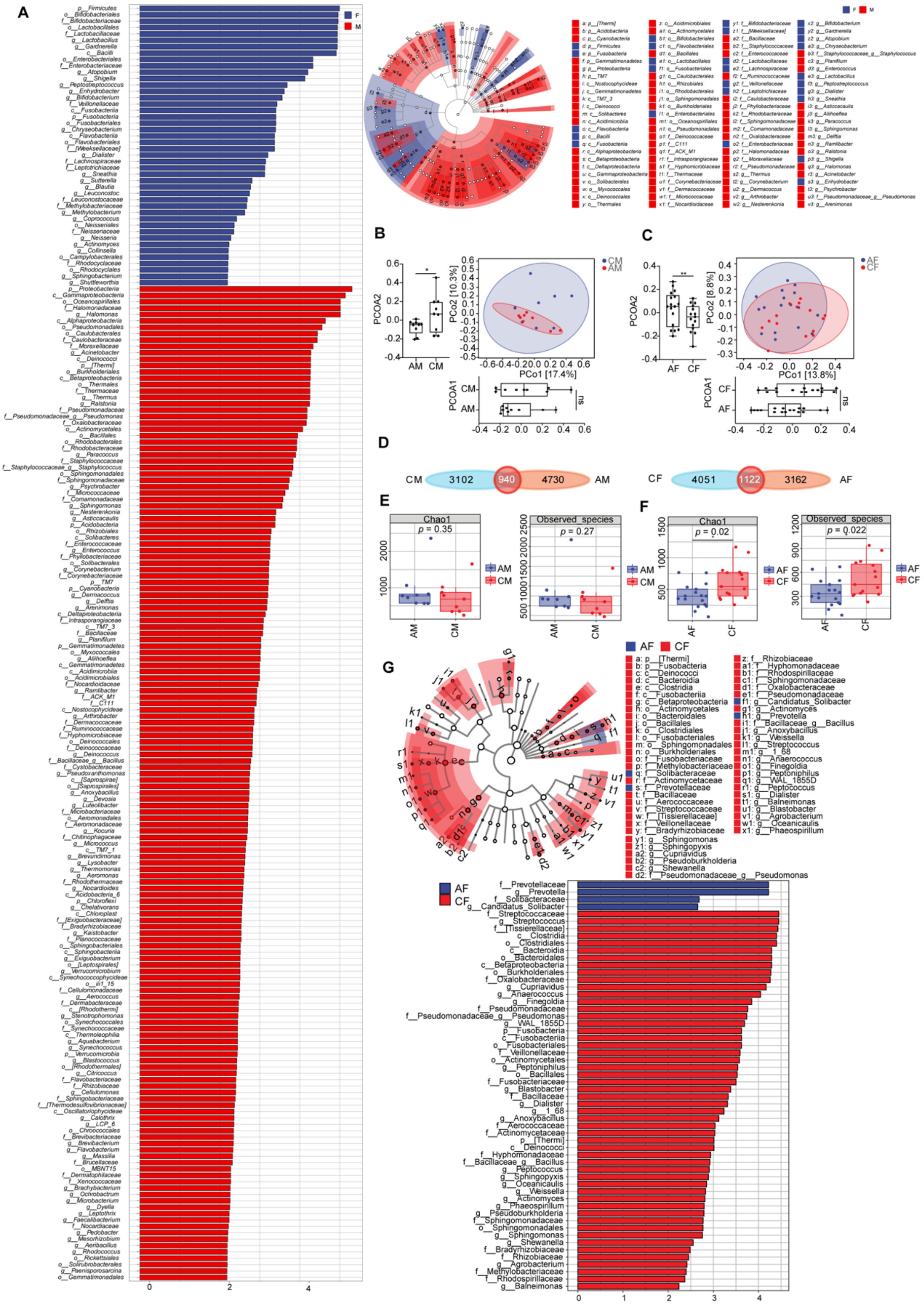

Figure S3. Characteristic sex-related differences in the urinary microbiome at the transitional stage of the age-related decline in renal function.

(A) LEfSe to identify potential species markers. A cladogram of taxonomic representations of

significant differences between male and female groups is shown. Coloured nodes from the inner ring to the outer ring represent taxa from the phylum to genus level. The two colours represent different taxa. LDA score histogram of features with significant differences between groups (right). The LDA score threshold for identifying features was 2.0.

(B-C) PCoA based on UniFrac distances. Each sample is represented by a dot. The blue and red circles represent the AM–CM and AF–CF groups, respectively. The box chart statistics show the different degrees of the horizontal and vertical coordinates respectively; ns, $P > 0.05$; *, $P < 0.05$; **, $P < 0.01$.

(D) Venn diagrams at the OTU level. The two colours represent different groups. The overlapping part represents the common OTUs between groups; the non-overlapping part represents the unique OTUs in each group.

(E-F) Alpha diversity indices for the AM–CM and AF–CF groups, including the Chao1 and observed-species indices; *, $P < 0.05$.

(G) LEfSe to identify potential species markers. Cladogram of taxonomic representations of significant differences between the AF and CF groups. Coloured nodes from the inner to the outer ring represent taxa from the phylum to genus levels. The two colours represent different taxa. The LDA score threshold for identifying features was 2.0.
